# Development of an automated machine learning-based risk prediction and decision support system for postoperative cubitus varus complicating pediatric lateral humeral condyle fracture

**DOI:** 10.3389/fsurg.2026.1836372

**Published:** 2026-07-30

**Authors:** Yu Wan, Kun Deng, Yang Yuan, Li Zhang, Cong Li, Haoqi Cai, Yufeng Wang

**Affiliations:** Shanghai Children’s Medical Center, Shanghai Jiao Tong University School of Medicine, Shanghai, China

**Keywords:** automated machine learning, clinical decision support system, cubitus varus, improved langEvin equation-based evolutionary, lateral humeral condyle fracture

## Abstract

**Purpose:**

Postoperative cubitus varus is a disabling complication of pediatric lateral humeral condyle fractures that impairs long-term elbow function. This study aimed to develop and validate the first Improved LangEvin Equation-based Evolutionary (ILEE) automated machine learning (AutoML) model and visual decision support system for preoperative prediction of this complication, to enable personalized risk stratification and targeted intervention.

**Methods:**

We conducted a retrospective cohort study of 330 children with lateral humeral condyle fractures treated between January 2005 and June 2022. An ILEE-optimized AutoML model was constructed and compared with the original LEE algorithm and 6 conventional machine learning models. Model performance was evaluated using area under the receiver operating characteristic curve (ROC-AUC), accuracy, sensitivity, and specificity. Key predictors were identified and interpreted via SHapley Additive exPlanations (SHAP) analysis, and a clinical decision support system was developed using MATLAB App Designer.

**Results:**

The ILEE algorithm outperformed all comparators in optimization stability and convergence speed. The AutoML model identified five key features for predicting cubitus varus and exhibited better prediction calibration performance compared to other models (ROC-AUC = 0.9557). SHAP analysis ranked the importance of these features as follows: obesity degree, preoperative timing, internal fixation time, fracture type, and external fixation time. The decision support system can generate real-time risk levels, predicted probabilities, and personalized clinical recommendations within 1 s.

**Conclusion:**

This study establishes the first ILEE-based AutoML model for predicting postoperative cubitus varus in pediatric lateral humeral condyle fractures, demonstrating competitive predictive performance. The user-friendly visual system enables rapid preoperative risk assessment, assisting clinicians in identify high-risk patients, optimize treatment plans, and potentially improving pediatric orthopedic outcomes.

## 1 Introduction

Lateral humeral condyle fracture is one of the most common traumatic elbow injuries in children, predominantly affecting those aged 3–10 years and accounting for 15%–20% of all pediatric elbow fractures (1, 2). Although surgery is the standard of care for displaced fractures to achieve anatomical reduction and joint integrity, postoperative cubitus varus remains the most prevalent and refractory complication, affecting 10%–30% of patients (3). This deformity causes cosmetic concerns and disrupts elbow biomechanics, leading to long-term sequelae such as limited mobility, chronic pain, and post-traumatic osteoarthritis (4, 5). These outcomes significantly impair limb function and psychological well-being, imposing a substantial burden on families and healthcare systems (6). Therefore, accurate and individualized preoperative risk stratification is critical to enable early targeted intervention and improve outcomes.

Existing studies have developed multivariate logistic regression models that identify the direction of displacement, time from injury to reduction (7), and type of fracture (8) as risk factors for post-traumatic cubitus varus, which provide a foundation for basic clinical risk assessment. However, these models rely on traditional statistical methods that assume linear relationships and fail to capture complex nonlinear interactions between clinical factors, resulting in limited predictive performance [area under the receiver operating characteristic curve (ROC-AUC) of these models ranges from only 0.70 to 0.85] that cannot meet the demands of precision medicine (9). In terms of study population coverage, most studies focus on supracondylar humeral fractures, leaving a critical gap in specialized predictive research for lateral humeral condyle fractures—a high-risk subtype with distinct pathological mechanisms—thus lacking targeted clinical guidance. Furthermore, these models lack rigorous external validation, and no accompanying visual decision support tools exist, which hinders their translation of research into clinical practice (10).

Automated Machine Learning (AutoML), a cutting-edge AI innovation, addresses these limitations by automating the entire model development pipeline—from data preprocessing to feature selection and hyperparameter optimization—significantly lowering technical barriers for clinical researchers (11). While AutoML has shown promise in other medical fields, its application in pediatric orthopedics remains at an early stage, with only a few exploratory studies reported. However, existing AutoML frameworks rely on traditional swarm intelligence algorithms, such as genetic algorithms and particle swarm optimization, which suffer from uneven population initialization, slow convergence, and a high risk of trapping in local optima. These limitations are exacerbated when processing high-dimensional, noisy, heterogeneous clinical data, restricting the performance and reliability of the resulting prediction models.

To address these critical clinical and technical gaps, we innovatively propose an AutoML framework based on the Improved Langevin Equation-based Evolutionary (ILEE) algorithm, which simultaneously optimizes feature selection and hyperparameters—overcoming the suboptimality of traditional sequential optimization. We further develop a user-friendly visual clinical decision support system that automatically generates individual risk assessments and targeted intervention recommendations. To our knowledge, this is the first study to apply an improved physics-inspired evolutionary AutoML framework to pediatric orthopedic prognosis and the first to develop a standardized, interpretable risk assessment tool for postoperative cubitus varus. This study provides an accurate, reliable tool for personalized risk stratification in pediatric lateral humeral condyle fractures and offers a valuable paradigm for AutoML applications in pediatric orthopedics.

## 2 Methods

### 2.1 Data collection

**Figure 1:**
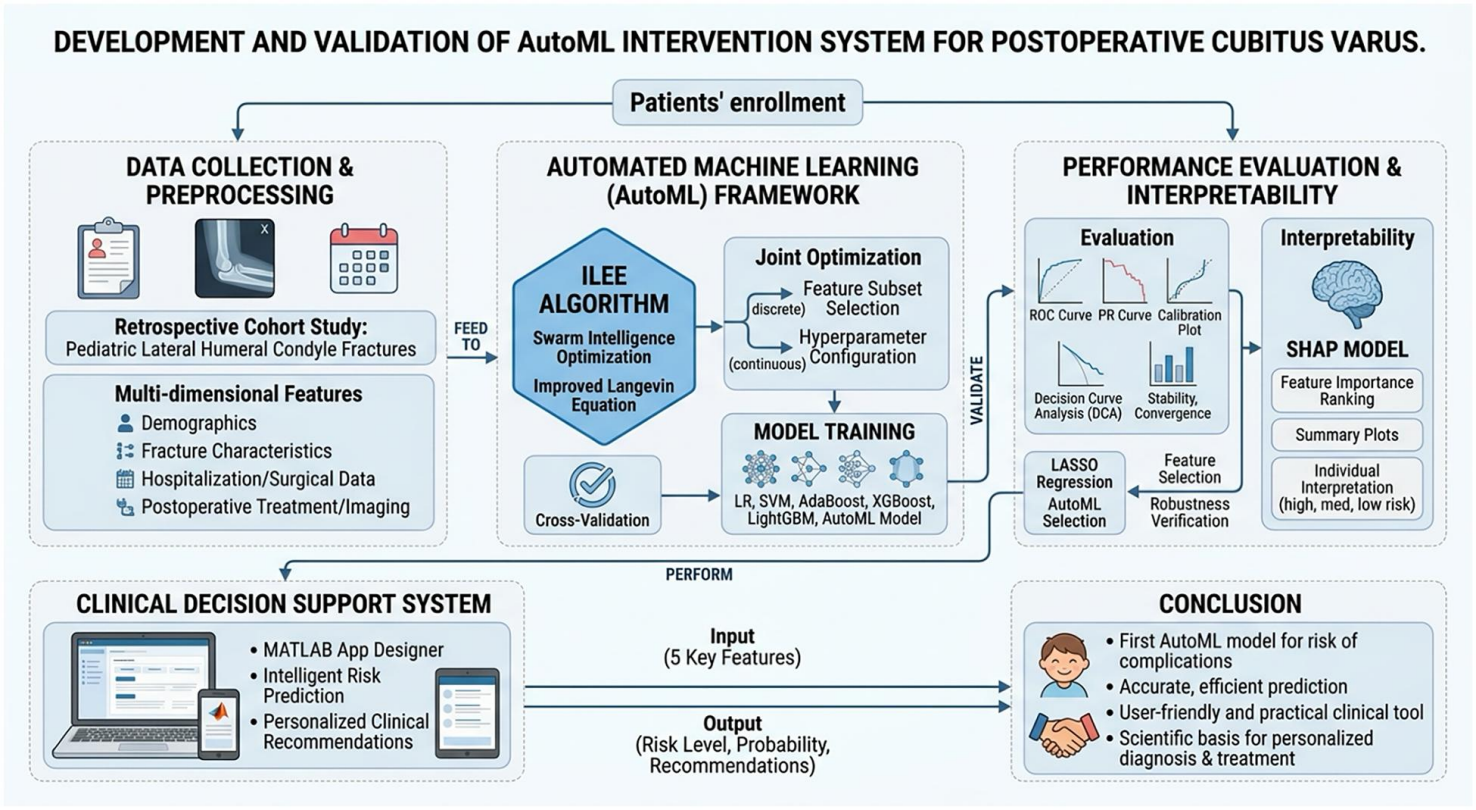
Study design.

This was a retrospective cohort study aimed at constructing a predictive model for the risk of postoperative cubitus varus in children with lateral humeral condyle fractures (Figure 1). Data were extracted from the medical records of children diagnosed with lateral humeral condyle fractures who were admitted to the Department of Pediatric Surgery of our hospital between January 2005 and May 2022.

Patients were eligible for inclusion in the trial if they were under 14 years old, diagnosed with a lateral humeral condyle fracture (11) and had complete case information. Patients with Open fractures, pathological fractures, multiple fractures, Jacob type I fractures (4) or combined with severe neurovascular injuries were excluded. Patients whose Baumann angle of the affected limb exceeded the normal range (70°–75°) on the anteroposterior x-ray film on the day after surgery were also excluded.

Data collection was completed by reviewing the hospital's electronic medical record management system and picture archiving and communication system. The collected clinical features covered preoperative, intraoperative, and postoperative periods, as detailed below:

(1) Demographic characteristics: Including the patient's age, gender, and weight. Obesity degree was calculated and classified (normal weight, mild obesity, moderate obesity, severe obesity) based on age and standard weight formulas.
(2) Fracture and preoperative characteristics: Including fracture type (classified as Jacob type II or III according to Jacob classification) and timing of surgery (divided into fresh fractures and old fractures with 21 days from injury to surgery as the cutoff).
(3) Hospitalization and surgical characteristics: Including days of hospital stay, surgical approach (closed reduction percutaneous Pin fixation, open reduction screw internal fixation, open reduction Pin fixation), operation time, and intraoperative blood loss.
(4) Postoperative treatment and imaging characteristics: Including internal fixation time (number of days from implantation to removal of internal fixation), external fixation time (number of days from removal of internal fixation to removal of external fixation), and Baumann angle measured on the anteroposterior x-ray film of the elbow joint on the day after surgery.

The outcome measure was defined as the carrying angle of affected limb 1 year postoperatively. It was measured by rechecking the standard anteroposterior x-ray film of the elbow joint, and the angle between the humeral axis and the ulnar axis was independently evaluated by two radiologists blinded to the clinical data. According to clinical consensus (11, 12), a carrying angle less than 0° was defined as cubitus varus. To assess the reliability and consistency of radiographic measurements, this study conducted an interobserver reliability analysis for the key continuous radiographic variable—the Baumann angle measured on anteroposterior elbow radiographs taken on the first postoperative day. All measurements were performed independently by two radiologists who were blinded to the clinical data and grouping information. We used the intraclass correlation coefficient (ICC) with a two-way random effects model to quantify the agreement between the two raters’ measurements. Generally, an ICC value between 0.75 and 0.90 indicates good agreement, and a value above 0.90 indicates excellent agreement. After calculation, the interobserver ICC for the Baumann angle in this study was 0.92 (95% CI: 0.89–0.94), demonstrating excellent reproducibility of the measurement, thereby ensuring the objectivity and reliability of the radiographic data in this study. This study was reviewed and approved by the Ethics Committee of our hospital.

### 2.2 Automated machine learning framework

#### 2.2.1 Swarm intelligence-based joint optimization

This study presents an automated machine learning framework that integrates swarm intelligence algorithms to simultaneously optimize feature subset selection and hyperparameter configuration through a unified search process. Unlike traditional sequential optimization approaches that treat feature selection and hyperparameter tuning as independent stages, our methodology employs a joint optimization paradigm. In this paradigm， each candidate solution in the swarm represents a complete configuration comprising both discrete feature subsets and continuous hyperparameter values.

The optimization process is driven by swarm intelligence algorithms, which simulate collective behaviors observed in natural systems to solve complex optimization problems. The foundational Langevin equation evolution (LEE) algorithm serves as the core optimization engine (13). This algorithm is inspired by stochastic dynamics in physical systems, specifically modeling the random motion patterns of microscopic particles under thermal fluctuations. The LEE algorithm captures the essence of Brownian motion, where particles exhibit probabilistic trajectories governed by both deterministic forces and random perturbations. The algorithm's optimization mechanism consists of three key components: a global search strategy based on Langevin stochastic differential equations that enables extensive exploration of the solution space, a thermal fluctuation balance mechanism that maintains population diversity by simulating energy exchange in thermal systems, and a barrier-crossing mechanism that allows particles to overcome local energy barriers and escape from suboptimal regions.

To enhance the optimization performance for our specific application, we developed an ILEE algorithm with two critical enhancements. This algorithm introduces two key improvements. First, chaotic mapping is adopted for population initialization to replace the original random initialization method. Specifically, we utilize the Logistic chaotic map $x_{n+1}=\mu x_{n}(1\;-\;x_{n})$ to generate an initial solution sequence with uniform distribution and inherent pseudorandomness, and map the chaotic variables to the search space, thereby enhancing the ergodicity and coverage uniformity of the initial population in the solution space and laying a solid foundation for subsequent global search. Second, an adaptive Lévy flight step size strategy is designed. The Lévy flight is a random walk pattern that follows a power-law distribution, where the step length *s* is generated by the formula $s\;∼\;u\;/\;|v|\hat{\left\{\frac{1}{\beta }\right\}}$, with *u* and *v* following normal distributions and β controlling the tail characteristics of the distribution. In each iteration of the ILEE algorithm, an adaptive factor is dynamically calculated based on the current iteration number t and the maximum number of iterations *T*. This factor decreases nonlinearly with the iteration process and is used to modulate the step length of the Lévy flight. In the early stage of optimization, a larger step length drives the algorithm to perform large-scale, long-jump global exploration; in the later stage, the step length gradually decreases, allowing the algorithm to focus on fine exploitation of local regions. This mechanism enables the ILEE algorithm to autonomously achieve a smooth transition between exploring unknown regions and mining known optimal regions, effectively improving the convergence speed and the ability to escape local optima. The algorithmic improvements were validated using the CEC2022 benchmark test functions (14), demonstrating superior convergence speed and solution quality compared to the original LEE algorithm. To more clearly elucidate the collaborative workflow between the ILEE algorithm and the AutoML framework it drives, the pseudocode of the core steps is provided below (Supplementary Material 1).

#### 2.2.2 Synchronous optimization architecture

In our AutoML framework, each individual in the swarm population encodes a complete solution vector that simultaneously specifies: (1) a binary feature mask indicating which predictors are selected (discrete component), and (2) a set of continuous hyperparameter values for the machine learning model (continuous component). During each iteration, the swarm intelligence algorithm evaluates the fitness of each candidate solution by training a model with the specified feature subset and hyperparameters, then assessing its cross-validated performance. The algorithm then updates the population positions based on the collective intelligence mechanism, where individuals share information about promising regions in the joint search space. Through iterative evolution, the swarm converges toward optimal combinations of features and hyperparameters that maximize predictive performance.

Six comparative models were established in this study: Logistic Regression (LR), Support Vector Machine (SVM), Adaptive Boosting (AdaBoost), Extreme Gradient Boosting (XGBoost), Light Gradient Boosting Machine (LightGBM), and the proposed AutoML framework, all implemented on the MATLAB 2024b platform. To ensure fair evaluation and reproducibility, the dataset was first randomly partitioned into training and testing sets at a 7:3 ratio, with stratification applied to maintain class distribution consistency. This partitioning and all subsequent stochastic operations were strictly controlled by fixing the MATLAB random number generator seed to rng (42). After this split, all continuous input variables underwent Z-score normalization, transforming their distributions to have a zero mean and unit standard deviation. Crucially, the mean and standard deviation parameters required for this standardization were computed solely from the training set data and were subsequently applied uniformly to the test set, thereby preventing information leakage. Following standardization, five-fold cross-validation was employed on the training set to obtain robust performance estimates and mitigate overfitting. To address the class imbalance problem in the training set, the Synthetic Minority Over-sampling Technique was adopted for data augmentation. During SMOTE implementation, the key parameter settings were as follows: the number of nearest neighbors k was set to the default value of 5; the oversampling target was set to achieve a balanced 1:1 distribution between the minority class samples (i.e., cases with postoperative cubitus varus) and the majority class samples in the training set. By constructing models within this fully traceable preprocessing framework, we can ensure the fairness of model performance evaluation and the reliable reproduction of results.

### 2.3 Performance metrics

Model performance was evaluated using a comprehensive set of metrics that assess classification accuracy, probability calibration, and clinical decision-making utility. Classification performance was measured through six complementary indicators: Accuracy (ACC) quantifies the proportion of correctly classified cases; Sensitivity (SEN) represents the true positive rate, indicating the model's ability to identify patients at risk; Specificity (SPE) measures the true negative rate, reflecting correct identification of low-risk patients; F1-score provides a harmonic mean of precision and recall, offering a balanced assessment when class distributions are uneven; ROC-AUC summarizes the model's discriminative ability across all possible classification thresholds, with values ranging from 0.5 (random performance) to 1.0 (perfect discrimination); and Area Under the Precision-Recall Curve (PR-AUC) is more sensitive for evaluating model performance on imbalanced datasets by focusing on the precision-recall trade-off.

Beyond classification metrics, we evaluated the reliability of predicted probabilities through calibration analysis. Calibration curves were constructed by dividing predicted probabilities into bins and plotting the mean predicted probability against the observed event rate within each bin. Perfect calibration occurs when predicted probabilities align with observed frequencies across all probability ranges. The Brier score, computed as the mean squared difference between predicted probabilities and actual binary outcomes, provides a quantitative measure of calibration quality, with lower scores indicating superior probabilistic accuracy.

To assess clinical applicability, Decision Curve Analysis (DCA) was employed to quantify the net benefit of model-guided clinical decisions across a spectrum of threshold probabilities. The net benefit calculation incorporates both the clinical value of true positives and the cost of false positives, expressed as: Net Benefit = (True Positives/Total Patients) - (False Positives/Total Patients) × [Threshold Probability/(1 - Threshold Probability)]. By comparing the net benefit of the model against default strategies (treating all patients or treating none), DCA identifies the range of threshold probabilities where the model provides superior clinical utility, thereby guiding practical implementation decisions.

### 2.4 Interpretability analysis

To comprehensively evaluate prognostic predictors and ensure methodological rigor, this study implemented a multi-phase analytical framework that integrates automated feature selection with independent validation and detailed interpretability assessment. Initially, an automated machine learning (AutoML) approach was employed to systematically identify feature subsets associated with clinical outcomes based on predefined optimization criteria within a specified search space. Following this, to independently assess the consistency and validity of the AutoML-derived features, LASSO regression (Least Absolute Shrinkage and Selection Operator) was applied directly to the entire dataset without any prior filtering, generating a separate feature subset for comparative analysis. The degree of overlap between the features selected by AutoML and those identified through LASSO was then quantitatively examined, providing a measure of agreement that helped validate the robustness and rationality of the AutoML-based selection process. Subsequently, the SHAP (SHapley Additive exPlanations) framework, grounded in cooperative game theory principles, was utilized to enhance model transparency and clinical interpretability. This involved calculating Shapley values to numerically attribute feature contributions to individual predictions, while summary plots were generated to reveal global feature importance rankings. Additionally, waterfall plots, decision path plots, and force plots were employed to visually depict the interpretation process for specific cases, thereby offering an intuitive understanding of how selected features influence prognostic outcomes. This integrated methodology not only strengthens the reliability of feature selection through cross-validation but also ensures that the model's logic remains clinically plausible and mathematically interpretable.

### 2.5 Clinical decision support system

A user-friendly clinical decision support application was developed using MATLAB 2024a App Designer, integrating the trained machine learning models into an interactive graphical interface. The software enables real-time risk assessment by accepting patient clinical and imaging parameters as inputs, generating probability estimates for postoperative cubitus varus development, and presenting evidence-based clinical recommendations. The interface was designed following human-computer interaction principles to minimize cognitive load and facilitate rapid clinical decision-making.

### 2.6 Statistical analysis

All study data were uniformly processed and imported into SPSS 26.0 software for standardized statistical analysis. Continuous variables following a normal distribution were reported as mean ± standard deviation (x¯ ± s), while those with non-normal distribution were expressed as median [interquartile range, M (Q1, Q3)]. Categorical variables were summarized by frequency and percentage (n, %).

Between-group comparisons were performed using appropriate statistical methods: normality tests were first conducted for continuous variables; independent-samples t-test was adopted if both groups were normally distributed, and Mann–Whitney U test was used for non-normally distributed data. Pearson chi-square test was applied for the comparison of categorical variables. All statistical tests were two-sided, with a significance level defined as *α* = 0.05, and the findings of this study were systematically presented in tabular format.

## 3 Result

### 3.1 Patients

According to the inclusion and exclusion criteria, a total of 330 children were finally enrolled. All children underwent surgical treatment, with a mean age of (6.08 ± 3.00) years, including 228 males (69.09%) and 102 females (30.91%). There were no statistically significant differences in all baseline characteristics between the training set (*n* = 231) and the test set (*n* = 99) (all *P* > 0.05), indicating the effectiveness of stratified random sampling: the proportion of cubitus varus was highly consistent between the two groups (18.18% in the training set vs 19.19% in the test set, *χ*^2^ = 0.047, *P* = 0.829) (Table 1).

**Table 1:** Characteristics of the patients at baseline.

Characteristic	Training set (*n* = 231)	Test set (*n* = 99)	t/*χ*^2^/z	P value
Outcome measure				
Cubitus varus, n (%)	42 (18.18)	19 (19.19)	0.047	0.829
Demographic factors				
Age(years)	6.12 ± 3.05	5.98 ± 2.91	0.387	0.699
Male, n (%)	160 (69.26)	68 (68.69)	0.011	0.917
Fracture and preoperative				
Fracture type, n (%)			0.023	0.880
Jacob II	185 (80.09)	80 (80.81)		
Jacob III	46 (19.91)	19 (19.19)		
Timing of surgery, n (%)			0.224	0.636
Fresh fractures	201 (87.01)	88 (88.89)		
Old fractures	30 (12.99)	11 (11.11)		
Obesity, n (%)			0.775	0.855
Normal	21 (9.09)	8 (8.08)		
Mild obesity	163 (70.56)	68 (68.69)		
Moderate obesity	25 (10.82)	14 (14.14)		
Severe obesity	22 (9.52)	9 (9.09)		
Surgical factors				
Surgical approach, n (%)			0.507	0.776
Closed reduction percutaneous Pin fixation	45 (19.48)	22 (22.22)		
Open reduction screw internal fixation	28 (12.12)	10 (10.10)		
Open reduction Pin fixation	158 (68.40)	67 (67.68)		
Intraoperative blood loss (mL)	8.00 [5.00, 10.00]	8.00 [5.00, 10.00]	0.175	0.861
Postoperative treatment and imaging factors				
Baumann angle (°)	72.45 ± 1.82	72.38 ± 1.75	0.324	0.746
Internal fixation time (days)	33.00 [30.00, 36.00]	33.00 [30.00, 35.00]	0.658	0.511
External fixation time(days)	10.00 [10.00, 10.00]	10.00 [10.00, 10.00]	0.505	0.614

### 3.2 Simulation performance test of algorithm improvement

**Figure 2:**
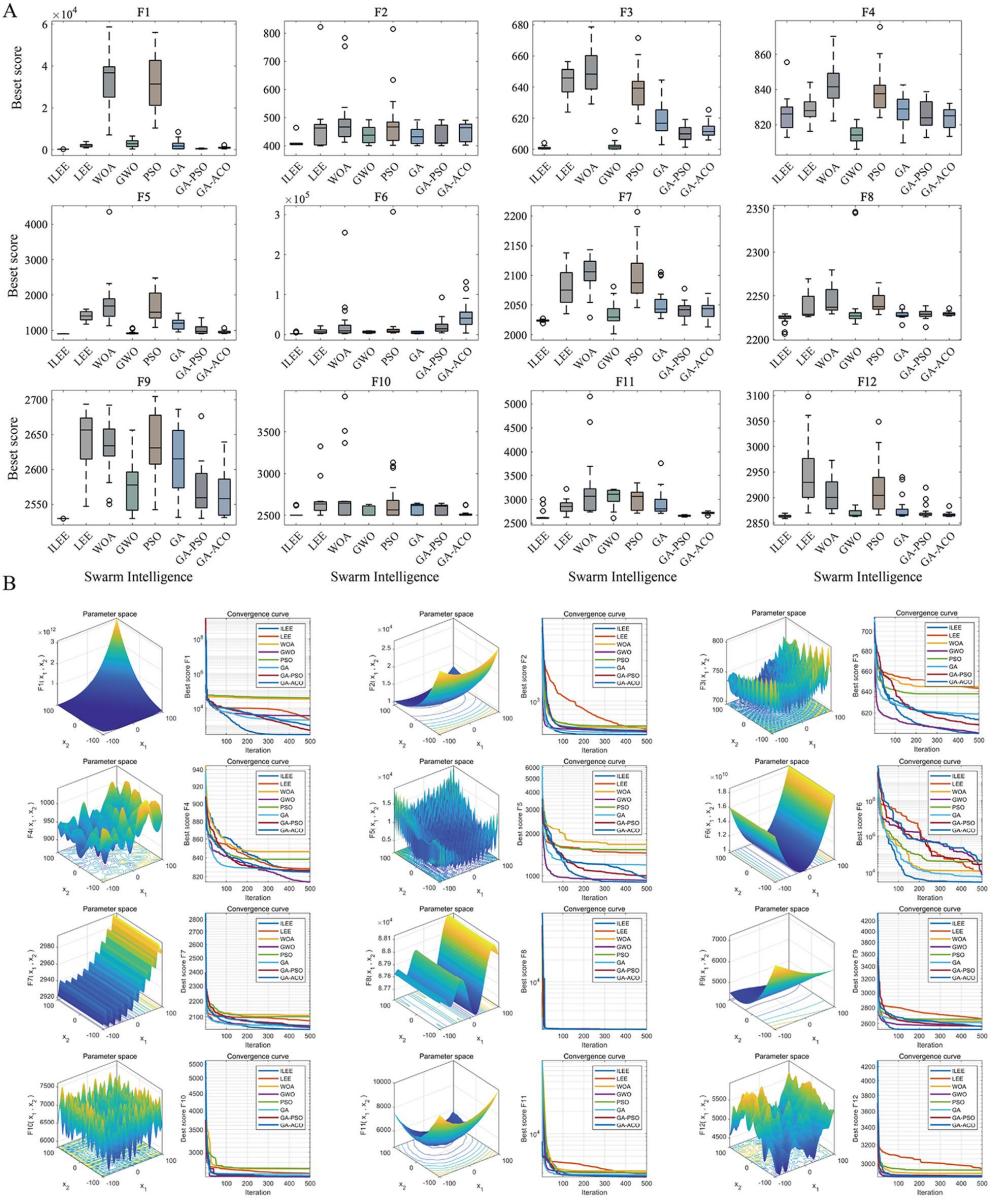
**Simulation test of improved swarm intelligence algorithms**. **(A)** Box plots of optimization results of each algorithm after 30 independent runs on CEC2022 benchmark functions, showing the optimization stability and robustness of each algorithm; **(B)** convergence curves of each algorithm during the optimization process, reflecting their convergence speed and ability to avoid local optima.

To verify the optimization ability of the improved ILEE algorithm, this study conducted comparative tests with the original LEE, WOA, GWO, PSO, GA, GA-PSO, and GA-ACO algorithms. All 12 benchmark functions from the CEC2022 test suite were adopted in the experiments. For all test functions, the variable dimension was set to 10, the population size to 30, and the maximum number of iterations to 500. Each algorithm was run independently 30 times to ensure statistical reliability. Box plots were plotted by comparing the results of 30 runs to evaluate the optimization stability of each algorithm. The results showed that the ILEE algorithm performed excellently on most benchmark functions, and its stability was significantly superior to that of the original LEE and other comparative algorithms (Figure 2A, Supplementary Material 2). Further analysis of the convergence curves indicated that the ILEE algorithm had a faster convergence speed and the lowest risk of falling into local optima during the iteration process (Figure 2B). The above experimental results fully confirm the significant advantages of the ILEE algorithm in terms of global optimization performance and convergence efficiency.

### 3.3 Model training results

**Figure 3:**
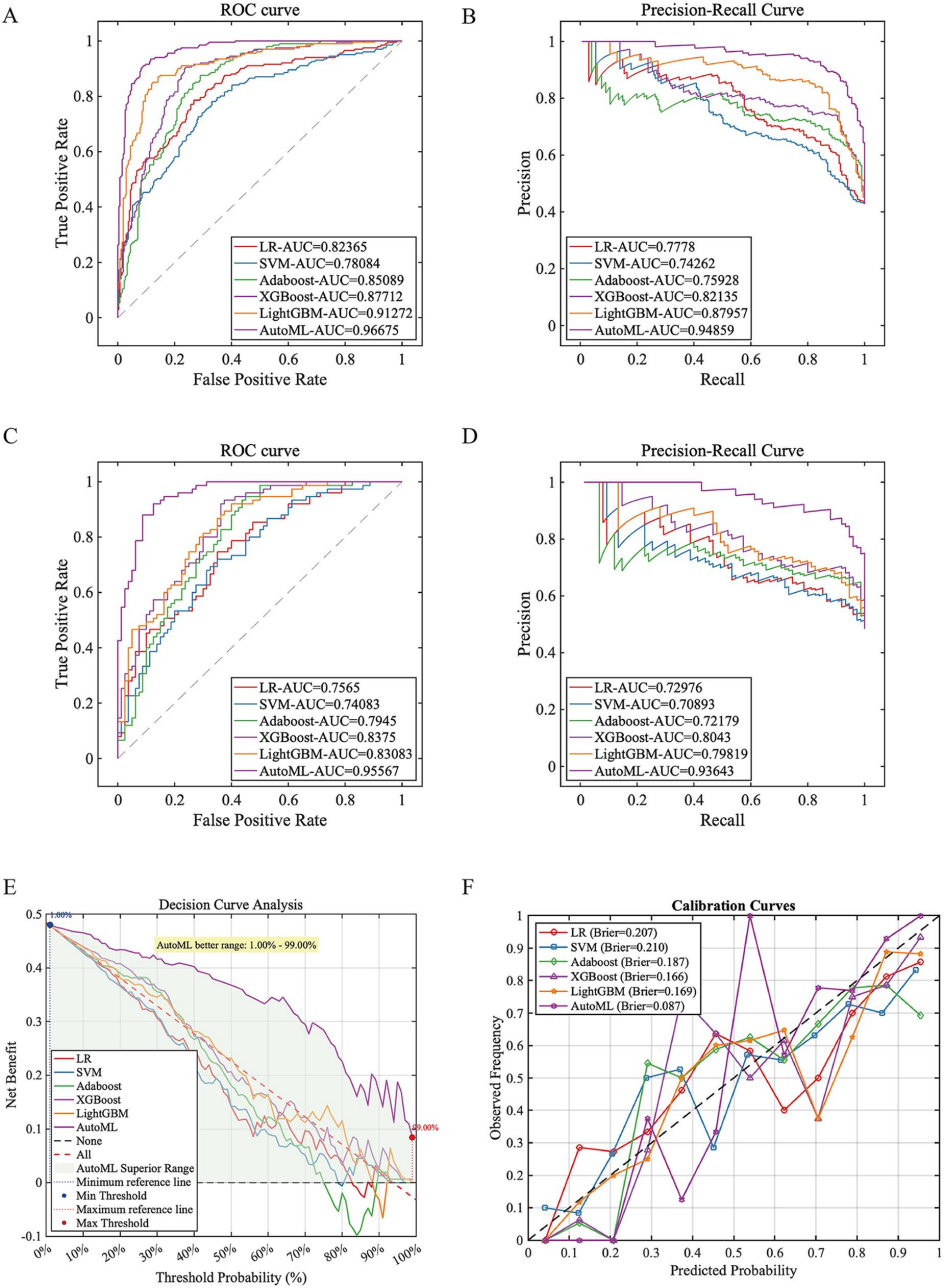
Training Status and performance verification of the prediction models. **(A)** ROC curve of the training set; **(B)** PR curve of the training set; **(C)** ROC curve of the test set; **(D)** PR curve of the test set; **(E)** DCA curve of the test set; **(F)** Calibration curve of the test set.

This study systematically evaluated the predictive performance of 6 machine learning models on the training set, with metrics including precision, sensitivity, specificity, accuracy, F1-score, and AUC. The results showed that the AutoML model exhibited the comprehensively optimal performance, with a ROC-AUC of 0.9668 and a PR-AUC of 0.9486 (Table 2, Figures 3A,B). Notably, the AutoML model had a particularly prominent advantage in F1-score (0.8688), indicating that it has stronger clinical application value in balancing precision and recall. The features finally screened by AutoML were: internal fixation time, preoperative timing, obesity degree, fracture type, and external fixation time.

**Table 2:** Performance evaluation indicators.

Data	Model	PRE	SEN	SPE	ACC	F1	ROC-AUC	PR-AUC
Training set	LR	0.6341	0.8706	0.6245	0.7298	0.7338	0.8237	0.7778
	SVM	0.5845	0.8607	0.5428	0.6787	0.6962	0.7808	0.7426
	Adaboost	0.6436	0.9254	0.6171	0.7489	0.7592	0.8509	0.7593
	XGBoost	0.6355	0.9453	0.5948	0.7447	0.7600	0.8771	0.8214
	LightGBM	0.6573	0.9353	0.6357	0.7638	0.7721	0.9127	0.8796
	AutoML	0.7967	0.9552	0.8178	0.8766	0.8688	0.9668	0.9486
Test set	LR	0.6200	0.8500	0.5000	0.6800	0.7170	0.7565	0.7298
	SVM	0.5600	0.8400	0.3000	0.6200	0.6720	0.7408	0.7089
	Adaboost	0.6400	0.9000	0.6000	0.7400	0.7481	0.7945	0.7218
	XGBoost	0.6600	0.8533	0.6375	0.7419	0.7443	0.8375	0.8043
	LightGBM	0.6400	0.9200	0.5000	0.7000	0.7549	0.8308	0.7982
	AutoML	0.7823	0.9500	0.7750	0.8600	0.8578	0.9557	0.9364

The performance of the models on the test set was evaluated, and the results showed that the AutoML model demonstrated the strongest robustness in the independent test set, with a ROC-AUC of 0.9557 and a PR-AUC of 0.9364 (Figures 3C,D); Decision curve analysis (Figure 3E) showed that the application of the AutoML prediction model in the test set within the risk threshold range of 1% to 99% could bring greater clinical net benefit compared with traditional methods. The net benefit curve of this model maintained a high level and remained stable over a wide range of threshold probabilities, indicating that it has good generalization ability and stable predictive performance; Calibration curve analysis (Figure 3F) confirmed that the predictive calibration performance of the AutoML model was significantly superior to other models, with the lowest Brier score (0.087) on the test set.

To further quantify whether the performance advantage of the AutoML model over other models is statistically significant, we conducted pairwise comparisons of the ROC-AUC for each model on the independent test set. The DeLong test was employed, and the AUC difference along with its corresponding 95% CI and P-value were calculated. The results showed that the AutoML model achieved an AUC of 0.9557 on the test set, significantly outperforming the traditional Logistic Regression model (AUC difference: 0.1992, 95% CI: 0.1055–0.2728, *P* < 0.001), the Support Vector Machine model (AUC difference: 0.2149, 95% CI: 0.1247–0.3051, *P* < 0.001), and the AdaBoost model (AUC difference: 0.1612, 95% CI: 0.0822–0.2402, *P* < 0.001). Similarly, compared with complex gradient boosting-based models, the AutoML model still demonstrated a clear advantage, with its predictive performance significantly higher than that of the XGBoost model (AUC difference: 0.1182, 95% CI: 0.0547–0.1817, *P* = 0.0003) and the LightGBM model (AUC difference: 0.1249, 95% CI: 0.0571–0.1927, *P* = 0.0003). The P-values for all comparisons were far below 0.05, and none of the 95% confidence intervals crossed zero, indicating that the improvement in predictive performance achieved by the AutoML model built upon the ILEE algorithm is genuine and robust, rather than arising from random error. These results provide solid statistical evidence for selecting AutoML as the optimal predictive model, reinforcing its reliability in clinical applications.

To quantify the specific contribution of SMOTE to the performance of the AutoML model, we conducted an ablation experiment in which the AutoML model was trained with and without SMOTE, while keeping all other preprocessing steps and model hyperparameters unchanged, and subsequently evaluated on the same test set. The results of the ablation experiment showed that after applying SMOTE, the sensitivity of the AutoML model on the test set increased substantially from 0.6800 to 0.9500, the F1-score improved from 0.7200 to 0.8578, and the PR-AUC rose from 0.8000 to 0.9364; meanwhile, specificity only experienced a minor adjustment from 0.8200 to 0.7750, accuracy increased from 0.7300 to 0.8600, and the ROC-AUC increased from 0.8100 to 0.9557. These results indicate that SMOTE, by synthesizing minority class samples, effectively corrected the learning bias caused by class imbalance, enabling the model to substantially enhance its ability to identify high-risk children without materially impairing its discriminative capacity for the majority class. Therefore, SMOTE is confirmed to be a necessary and effective component in the AutoML modeling pipeline of this study.

### 3.4 Key influencing factors analysis

#### 3.4.1 LASSO regression

**Figure 4:**
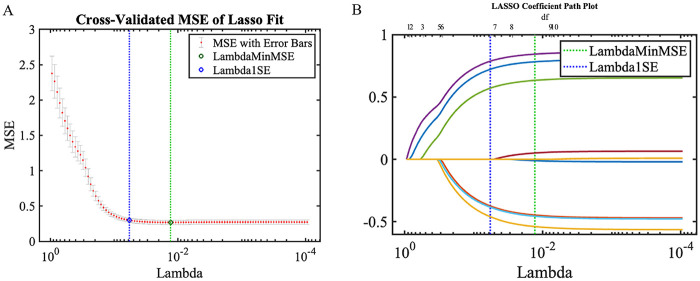
**LASSO result plots**. **(A)** LASSO trace plot; **(B)** LASSO cross-validation fitting plot.

LASSO regression was used for feature selection on the training set data (Figure 4) to verify the effectiveness of the features screened by the AutoML model. LASSO selects variables within one standard error of the minimum mean squared error (MSE) in the sparse model (Lambda1SE), and 6 variables were screened out, including all the features selected by AutoML.

#### 3.4.2 SHAP analysis

**Figure 5:**
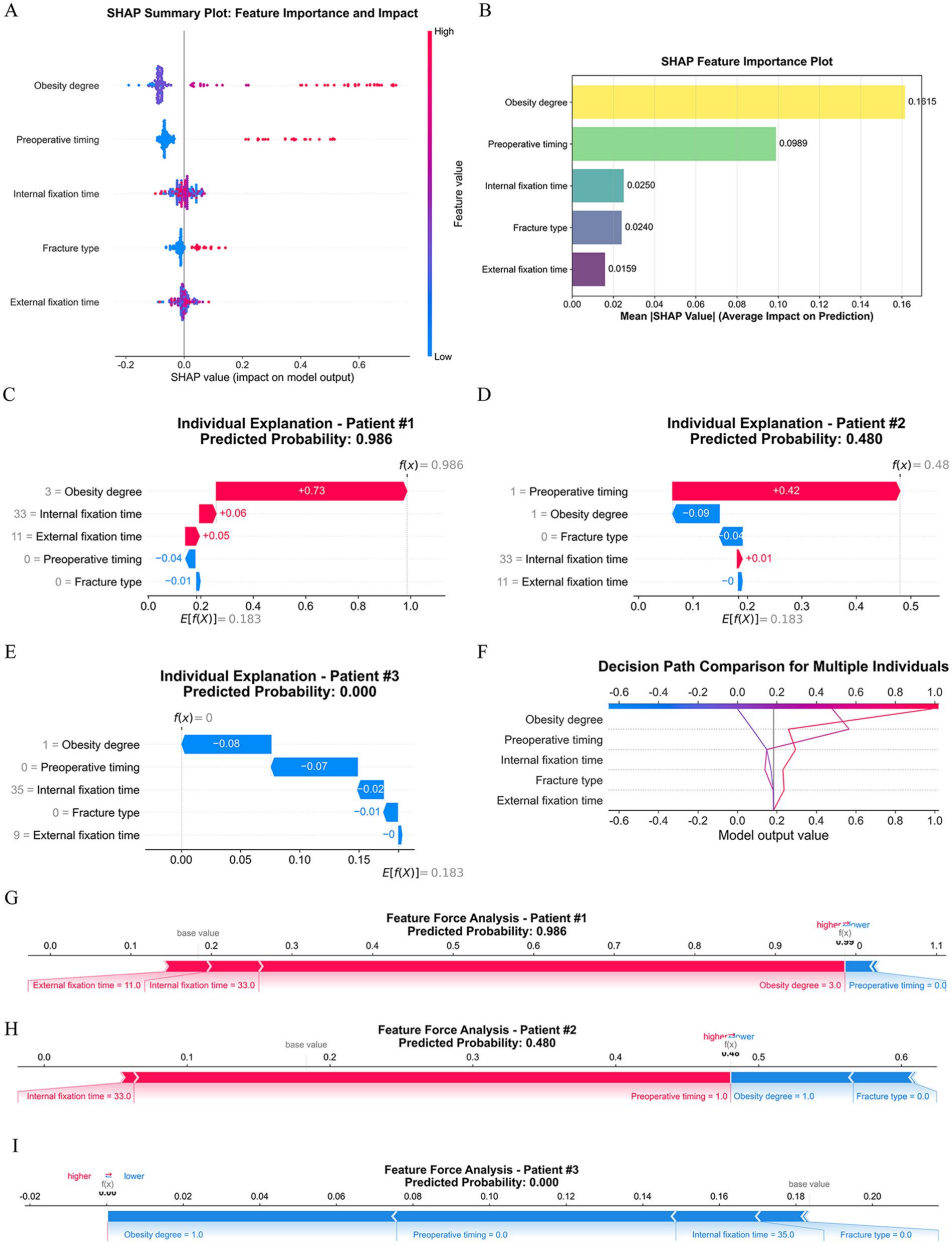
**SHAP interpretability analysis**. **(A)** shapley summary plot: shows overall feature impact on predictions (each point,  sample-feature SHAP value; color, feature value magnitude; horizontal axis, SHAP value direction/strength); **(B)** shapley feature importance plot (bar chart): ranks feature importance by mean absolute SHAP value (longer bars, greater influence); **(C,D,E)** waterfall plots: illustrate cumulative feature contributions to individual predictions (baseline, average prediction; red, risk increase; blue, risk decrease); **(F)** decision path plot: compares multi-patient decision pathways, showing feature combination effects on prediction outcomes; **(G,H,I)**: force plots: visualize feature-driven risk direction (red arrows, higher risk; blue arrows, lower risk; length, influence magnitude).

The SHAP analysis results (Figures 5A,B) showed that the feature importance ranking was as follows: obesity degree, preoperative timing, internal fixation time, fracture type, and external fixation time. By comparing the decision paths of patients with different risk levels (Figure 5F), systematic differences in feature combinations between high-risk and low-risk patients could be found. The paths of high-risk patients were significantly shifted to the right, indicating the combined effect of multiple high-risk features.

We selected one representative patient from each of the high-, medium-, and low-risk tiers and conducted an integrated interpretation of their individual prediction results using SHAP values. The high-risk patient had a predicted cubitus varus probability as high as 98.6%, with clinical features including fresh fracture, Jacob type II fracture, 33 days of internal fixation, and 11 days of external fixation; however, the SHAP contribution of severe obesity reached +0.73, acting as the absolutely dominant risk-driving force. Even though fresh fracture provided a protective effect of −0.04, it was far from sufficient to counteract this risk, while internal fixation time (+0.06) and external fixation time (+0.05) only slightly increased the probability. This suggests that for such patients, weight management should be the core intervention, and careful evaluation should be made regarding whether the fixation duration can be shortened to further reduce the risk. The medium-risk patient had a predicted probability of 48.0%, also with a Jacob type II fracture managed by 33 days of internal fixation and 11 days of external fixation, but the preoperative timing was an old fracture, whose SHAP value was as high as +0.42, constituting the primary risk factor. Although mild obesity (−0.09) and fracture type (−0.04) exhibited weak protective effects, they were still unable to suppress the risk brought by delayed surgery; therefore, early surgical treatment is the key to improving prognosis. The low-risk patient had a predicted probability of only 0.00%, with 35 days of internal fixation, 9 days of external fixation, fresh fracture, and mild obesity. All features showed negative SHAP values, among which mild obesity (−0.08) and preoperative timing (−0.07) synergistically produced a notable protective effect, while the influences of fracture type, internal fixation time, and external fixation time were near zero. This collectively formed an extremely low-risk pattern, and continuing routine follow-up without any special intervention is sufficient for such patients.

To further examine model behavior on failure cases, SHAP force plots were generated for representative misclassified samples in the test set (Supplementary Figure S1). In a representative false-positive case (internal fixation time, 32 days; chronic fracture; mild obesity; Jacob type III fracture; external fixation time, 10 days), the model assigned a predicted probability of 67.0% despite the absence of cubitus varus. The force plot indicated that preoperative timing (SHAP ≈ + 0.46) and fracture type (SHAP ≈ + 0.12) were the dominant features driving the incorrect prediction, as the combination of chronic fracture and Jacob type III fracture pushed the model toward the high-risk direction. By contrast, mild obesity (−0.05) and internal fixation time (−0.06) exerted protective effects but were insufficient to prevent misclassification. Unlike the typical high-risk pattern dominated by severe obesity, this case suggests that false-positive predictions may also occur when chronic fracture coexists with Jacob type III fracture. In a representative false-negative case (internal fixation time, 32 days; fresh fracture; moderate obesity; Jacob type II fracture; external fixation time, 11 days), the predicted probability was only 2.5% although cubitus varus actually occurred. Preoperative timing (−0.09), internal fixation time (−0.04), fracture type (−0.03), and external fixation time (−0.03) collectively pushed the prediction toward the low-risk direction, whereas obesity degree contributed only weakly (+0.02), leading the model to underestimate the true risk. These findings indicate that misclassifications were associated with conflicting feature signals and may help identify potentially confusing feature combinations for this classification task.

### 3.5 Construction of clinical decision support system

**Figure 6:**
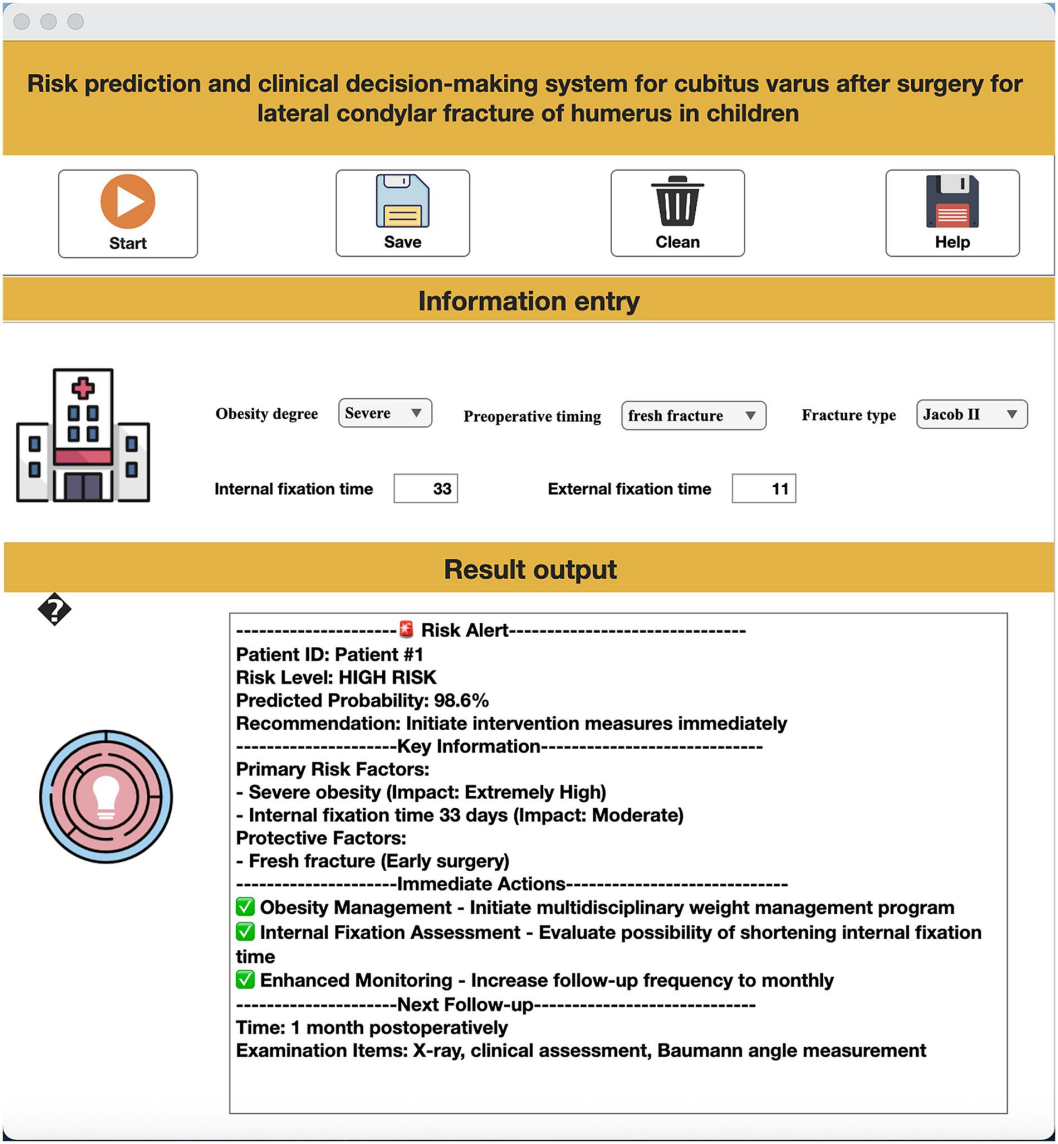
Demonstration of Decision Support System.

The above research has successfully identified the key features affecting patient outcomes. However, in practical clinical applications, the changes of these features are complex and intricate, making it difficult to intuitively reveal the prognostic risk of patients. Existing artificial intelligence methods have a high threshold for promotion and application, requiring clinical staff to have high programming skills and extensive literature knowledge, which makes it difficult for them to be popularized in general hospitals. To solve this problem, this study innovatively constructs a practical visualization system, which is built on the basis of selected key features and has the application advantages of intuitiveness, convenience and practicality. In the application process of the visualization system, users only need to input the specific values of 5 key features in the “feature input” column, and the system can automatically calculate the risk of cubitus varus and provide clinical suggestions (Figure 6).

## 4 Discussion

This study focuses on the clinically critical issue of cubitus varus after lateral humeral condyle fracture in children, and constructs an integrated risk prediction and decision support system that combines improved intelligent algorithms with interpretable machine learning. By deeply integrating the ILEE algorithm with AutoML, we systematically balanced the accuracy, stability, and clinical interpretability of the prediction model, providing a new paradigm for risk management of postoperative complications in pediatric orthopedics.

At the population inclusion level, this study strictly defined the inclusion criterion of age under 14 years based on three core lines of evidence: developmental anatomy shows that the elbow epiphyses are not yet closed at this stage, making children highly susceptible to epiphyseal plate injury and subsequent cubitus varus after lateral humeral condyle fracture; epidemiological data confirm that 4–10 years is the peak incidence age for this fracture type (9, 13); and excluding confounding factors such as open fractures and pathological fractures ensured the homogeneity of the study cohort. However, it should be noted that this rigid age limit may overlook individual developmental differences in some adolescents aged 13–14 years with accelerated skeletal maturation, whose fracture healing mechanisms may differ from younger children. Future studies should explore the impact of age stratification based on skeletal maturity rather than chronological age on model performance.

At the methodological level, the improved ILEE algorithm optimizes initial population distribution via chaotic mapping and implements an adaptive Lévy flight step size strategy, effectively solving the problems of insufficient spatial ergodicity and easy entrapment in local optima that plague traditional algorithms. More importantly, this study established a three-level interpretability framework consisting of “AutoML initial feature screening - LASSO regression independent validation - SHAP in-depth quantitative analysis”, breaking through the “black box” limitation of traditional machine learning and generating significant clinical practical value. First, the global feature importance ranking clarified the decision-making priority of core variables such as obesity degree and preoperative timing, providing precise targets for clinical intervention. Second, individual-level SHAP waterfall plots and decision path plots intuitively demonstrated the feature combination patterns of children with different risk levels, enabling clinicians to quickly identify key risk sources. Third, quantitative analysis of feature contributions revealed the magnitude of each factor's effect, providing a numerical basis for developing individualized intervention plans.

This study systematically validated the practical value of the SMOTE technique within the AutoML modeling pipeline through an ablation experiment. The incidence of cubitus varus in the training set was approximately 18%, and this naturally imbalanced distribution tends to bias the model toward the majority class, resulting in a model with high specificity but low sensitivity, which may lead to a substantial number of missed high-risk cases in clinical screening. The results of the ablation experiment clearly demonstrated that without SMOTE, the test set sensitivity of the AutoML model was only 0.6800 and the ROC-AUC was 0.8100, indicating a severely insufficient ability to identify events. After applying SMOTE to balance the positive-to-negative sample ratio in the training set to 1:1, sensitivity surged to 0.9500, ROC-AUC rose to 0.9557, and probability calibration also improved (PR-AUC increased from 0.8000 to 0.9364), while specificity remained at an acceptable 0.7750. This pattern of “substantially improved sensitivity with a mild decrease in specificity” is fully consistent with the “safety-first” principle in clinical screening, i.e., it is preferable to conservatively over-alert rather than to miss any child who may develop cubitus varus. It should be emphasized that SMOTE synthesized new samples exclusively within the training set, while the test set remained completely untouched in its original distribution. This rigorous protocol eliminated any potential information leakage at its source, ensuring the objectivity and fairness of the performance evaluation.

The results of feature analysis provide clear targets for clinical intervention, with all five key predictors identified by AutoML having well-established clinical pathological mechanisms. Previous studies have shown that obesity increases soft tissue tension around the joint (15), making anatomical reduction more challenging and raising the risk of postoperative fixation loosening. Compared with normal-weight children, the abnormal expression of adipokines and inflammatory factors in obese children can inhibit osteoblast activity through the OPG/RANKL pathway, leading to delayed callus maturation and insufficient mechanical strength (16). Kirschner wires of the same diameter bear greater mechanical stress at the fracture site in obese children, increasing the likelihood of postoperative micro-displacement and subsequent cubitus varus. Moreover, obesity is associated with poorer compliance and effectiveness of postoperative brace wearing (17), which may indirectly reduce the effective duration of external fixation and further increase the risk of cubitus varus. A study involving 107 children with humeral fractures confirmed this hypothesis (18). Consistent with these findings, our study identified obesity as a major driver of postoperative cubitus varus, and the pattern of “severe obesity + 33 days of internal fixation” observed in high-risk cases in the SHAP individual analysis is a true portrayal of the coexistence of excessive load and inadequate support.

The epiphyses on both ends of long bones in children are critical structures responsible for the longitudinal growth of the immature skeleton. Stimulation of the epiphysis by fractures or other factors can lead to epiphyseal overgrowth; theoretically, the greater the intensity and duration of the stimulus, the more pronounced the epiphyseal overgrowth. The Jacob classification (4) indicates that, compared with Jacob type II, Jacob type III fractures exhibit more significant rotation and displacement, as well as more severe soft tissue hinge injury. Even when anatomical reduction is achieved visually during surgery, residual micromotion and articular step-offs create a highly unstable mechanical basis in lateral column early in the postoperative period (19). Weiss et al. found that the incidence of cubitus varus for Jacob type II fractures was one-third that of Jacob type III, at 11% and 34% (20). Martins indicated that mild displacement of the distal fracture fragment during healing of lateral humeral condyle fractures is a cause of cubitus varus (21). Therefore, higher-grade fracture types are associated with an elevated risk of postoperative complications. Our study compared Jacob type II and Jacob type III fractures and found that cubitus varus was more likely to occur in Jacob type III fractures (mean SHAP ≈ 0.024). In addition, more severe fractures may compromise the stability of internal fixation. Traction from the common extensor tendon can then result in mild postoperative separation of the fracture fragment, with subsequent callus formation within the fracture gap, which also predisposes patients to cubitus varus (2).

Optimal preoperative timing should be selected to reduce the occurrence of postoperative complications, and most researchers believe that fracture prognosis is related to the length of time before surgery. A preoperative delay of more than 21 days is defined as an old fracture, at which point the hematoma at the fracture site has organized and fibrous connections have formed, and even malunion with callus may occur, making surgical exposure and separation difficult, precise reduction challenging, and internal fixation placement in the optimal bony channel difficult (22). This directly reduces the immediate holding power of the fixation system. Khare et al. treated 21 cases of old lateral humeral condyle fractures and found that surgical outcomes worsened with increasing surgical delay; all patients treated at 3–4 weeks after injury had excellent outcomes, whereas only 1 of the patients treated at 9–12 weeks had an excellent outcome (6, 23). Pathophysiological analysis of fracture healing indicates that old lateral humeral condyle fractures are characterized by fracture fragment resorption, rotational displacement, malunion, and extensor tendon contracture, which frequently preclude anatomical reduction intraoperatively. Furthermore, the compromised blood supply at the fracture site necessitates prolonged internal fixation to achieve adequate stability, which further stimulates epiphyseal overgrowth and consequently results in cubitus varus deformity (24). In our study, preoperative timing was the second most important predictor of cubitus varus after obesity degree; in mild obese children, old fractures even emerged as the dominant risk factor for this deformity. Therefore, in future clinical practice, we need to pay more attention to the follow-up of patients with Jacob type I fractures to prevent them from becoming old fractures.

Leonidou et al. prospectively followed 105 children with lateral humeral condyle fractures treated with open reduction and Kirschner wire internal fixation. Kirschner wires were removed once radiographic union was confirmed, with a mean union time of 33 days, and no cases of cubitus varus deformity were observed (25). Thomas et al. treated 104 children using the same surgical technique and concluded that 3 weeks of Kirschner wire fixation was sufficient for fracture union (26). Kirschner wires were removed at exactly 3 weeks postoperatively, and only one patient was removed at 19 days, resulting in delayed fracture healing, while all other patients did not develop cubitus varus. Consistently, a meta-analysis using a fixed-effects model demonstrated that shorter internal fixation time was associated with a lower incidence of postoperative cubitus varus and a higher rate of excellent and good elbow function (27). This indicates that prolonged internal fixation time is a risk factor for cubitus varus. The reason may be that the distal humeral fracture fragment contains an ossification center, which, when stimulated by the internal fixation device, can lead to overgrowth (28). The longer the internal fixation time, the stronger the stimulation to the epiphysis, and thus the more likely it is to cause cubitus varus (29).

In the present study, external fixation time serves primarily as a terminal protective measure. Following removal of internal fixation devices, external fixation acts as the final external constraint for maintaining coronal plane alignment of the elbow joint. While wearing external fixation braces, patients can perform appropriate isometric muscle exercises tailored to their individual conditions. Concurrently, the braces provide mechanical stability to maintain fracture alignment and exert a certain degree of biomechanical regulatory effect (9), which facilitates fracture healing. If external fixation is prematurely removed, the fracture fragment is exposed to unrestricted joint motion before sufficient healing strength has been achieved. Forearm gravity, muscle traction, and accidental loads during daily activities can then easily induce medial angulation (20, 30). Although the effects of external and internal fixation time on cubitus varus in our study were consistent with some previous reports, they did not play a decisive role in multidimensional risk assessment. This finding indicates that all relevant factors must be comprehensively considered during postoperative rehabilitation. Notably, obese children often have external fixation prematurely removed due to limited physical activity and parental overprotection, with the intention of “promoting joint mobility”. This human factor further exacerbates the vicious cycle among obesity, internal fixation time and external fixation time.

Obesity degree, fracture type, preoperative timing, and internal/external fixation time do not exert independent additive effects but rather form a sequential causal chain. Specifically, delayed surgery for higher-grade fractures frequently precludes optimal internal fixation stability or necessitates prolonged internal fixation. Postoperatively, obesity subjects the fracture site to greater mechanical stresses before adequate healing strength is achieved. Subsequent premature removal of external protection then renders cubitus varus highly likely.

Previous multivariate studies have predominantly focused on interactions between risk factors, such as how obesity and increased fracture severity synergistically contribute to prolonged internal fixation. In contrast, our study emphasizes the context-dependent independent effects of each risk factor across different pathological scenarios, a perspective that better aligns with the reality of interindividual variability in clinical practice. Our SHAP analysis revealed that identical clinical features exerted heterogeneous, and sometimes opposing, effects across different individuals. For instance, 33 days of internal fixation had a significantly greater impact on cubitus varus risk in severely obese children than in mildly obese children (SHAP value: +0.06 vs. + 0.01). Conversely, in children with fresh fractures and mild obesity, appropriately prolonged internal fixation exerted a slight protective effect (SHAP value: −0.02). Beyond confirming the dominant role of obesity, our study further demonstrated the dynamic shifts in variable contributions across different risk strata using decision path plots, providing a quantitative basis for precision interventions. In high-risk patients, even with seemingly acceptable fracture types and fixation durations, the presence of severe obesity alone contributed a positive SHAP value of +0.73, which was sufficient to outweigh the cumulative negative effects of all protective factors, resulting in a predicted probability as high as 98.6%. These findings underscore the importance of comprehensive risk assessment rather than isolated evaluation of individual factors, as the combined effect of multiple high-risk features typically far exceeds the simple sum of their individual effects.

The construction of the clinical decision support system has significantly enhanced the translational value of our research findings. Most existing artificial intelligence prediction models remain at the theoretical research level, with complex operations and poor interpretability that hinder widespread clinical application. Our MATLAB App Designer-based system encapsulates complex machine learning models into an intuitive tool: clinicians only need to input five key features to quickly obtain a patient's risk level, predicted probability, and targeted clinical recommendations. The system can not only identify high-risk patients (e.g., children with mild obesity combined with 33 days of internal fixation have a predicted risk of 48.0%) but also clarify core risk and protective factors, guiding interventions such as obesity management, optimization of internal fixation schemes, and adjustment of follow-up frequency.

This study has several specific limitations that should be acknowledged. First, this was a single-center retrospective study conducted at a tertiary children's hospital in Shanghai, which may introduce selection bias due to the relatively high proportion of severe cases referred to our center. Additionally, the study population was predominantly from Eastern China, where the childhood obesity rate is higher than the national average, which may affect the generalizability of the model to regions with different demographic characteristics. Although our sample size of 330 cases is larger than most previous studies on lateral condyle fractures, it is still insufficient to fully capture the rare but clinically important subgroup of patients with combined neurovascular injuries or complex fracture patterns. Second, we did not include several potentially critical features: quantitative indicators of fracture reduction quality, such as the gap and step-off between fracture fragments measured on postoperative CT scans, have been shown to be strong predictors of malunion (2); specific postoperative rehabilitation protocols, including the timing and intensity of elbow range-of-motion exercises, may affect joint alignment recovery; and nutritional status, particularly vitamin D and calcium levels, play a key role in bone healing. The absence of these variables may have limited the model's predictive accuracy. Third, our clinical decision support system is currently a MATLAB-based desktop prototype, which is not integrated with hospital electronic medical record systems. This may affect its operational efficiency in busy clinical settings, and its usability and acceptance among frontline clinicians have not been formally evaluated through user experience surveys. Finally, the model did not incorporate genetic factors, such as polymorphisms in genes related to bone formation and epiphyseal growth, which may explain some of the residual variability in fracture healing outcomes between patients with similar clinical characteristics.

Based on these limitations, future research will be conducted in the following targeted directions. First, a multi-center prospective cohort study will be initiated in collaboration with 5–7 tertiary children's hospitals across different regions of China, aiming to enroll 1,000–1,500 patients with lateral humeral condyle fractures. This will allow us to validate the external generalizability of our model and recalibrate it for different patient populations. Long-term follow-up of at least 2 years will also be conducted to assess the impact of early intervention guided by our system on long-term elbow function and growth. Second, we will enrich the feature set by including quantitative CT-based fracture reduction metrics, standardized postoperative rehabilitation records, and laboratory measurements of nutritional status. Advanced imaging techniques such as 3D motion capture will also be used to collect dynamic elbow function data during rehabilitation, which will further improve the model's predictive performance. Third, the decision support system will be redeveloped as a web-based application integrated with hospital electronic medical record systems, enabling automatic extraction of patient data and real-time risk assessment. A randomized controlled trial will then be conducted to evaluate the clinical effectiveness of the system, comparing the incidence of postoperative cubitus varus and patient outcomes between the intervention group (using the system) and the control group (standard care). Finally, we will explore the role of genetic factors in fracture healing by conducting a targeted gene association study in a subset of patients, focusing on genes related to bone metabolism and epiphyseal growth, with the goal of developing a more comprehensive multi-omics prediction model.

## 5 Conclusion

The AutoML prediction model based on the ILEE algorithm constructed in this study achieves accurate prediction of the risk of cubitus varus after lateral humeral condyle fracture in children, clarifies the core influencing factors, and the established visual decision support system provides clinicians with an intuitive and easy-to-use tool. This study not only provides a scientific basis for the individualized diagnosis and treatment of lateral humeral condyle fractures in children, but also offers valuable insights for the application of machine learning technology in the field of pediatric orthopedics. It is expected that through early risk identification and precise intervention, the incidence of postoperative cubitus varus can be reduced, and the clinical outcomes of children can be improved.

## Data Availability

The raw data supporting the conclusions of this article will be made available by the authors, without undue reservation.
