# Supplementary material for: Development of an automated machine learning-based risk prediction and decision support system for postoperative cubitus varus complicating pediatric lateral humeral condyle fracture
Source: Front Surg. 2026 Jul 30;13:1836372. doi: 10.3389/fsurg.2026.1836372 (PMC13469642; doi:10.3389/fsurg.2026.1836372)
Supplement: Supplementary file 1 [file Supplementaryfile1.docx]

**Supplementary materials 1**

**Pseudocode of the ILEE‑driven AutoML framework**

% Input: Dataset Data, population size N, maximum iterations MaxIter

% Parameters: friction coefficient γ, Lévy index β, cooling factor Cooling_Factor

% Output: Optimal feature subset F_best, optimal hyperparameters H_best

% 1. Initialization

Initialize population P of N particles via Logistic chaotic mapping;

Encode each position vector x_i as (binary feature mask, continuous hyperparameters);

Calculate fitness f_i = CV_Score(Data(x_i), Model(x_i));

Set personal best pbest_i = x_i and global best gbest;

Initialize temperature Temp = T0;

% 2. Iterative optimization

FOR t = 1 TO MaxIter

Compute adaptive Lévy flight step-scale factor α(t) based on t;

FOR i = 1 TO N

% Update rule based on Langevin equation

Compute deterministic drift Drift_i toward pbest_i and gbest;

Generate random step R_i = α(t) * Levy(β);

Diffusion_i = sqrt(2 * γ * Temp) * R_i;

x_i(t+1) = x_i(t) + Drift_i + Diffusion_i;

% Boundary handling and fitness evaluation

Clamp continuous part of x_i(t+1) to feasible bounds;

Binarize feature mask part of x_i(t+1);

f_new = CV_Score(Data(x_i(t+1)), Model(x_i(t+1)));

% Update personal and global bests

IF f_new is better than f(pbest_i)

pbest_i = x_i(t+1);

END IF

IF f_new is better than f(gbest)

gbest = x_i(t+1);

END IF

END FOR

% Anneal temperature to balance exploration and exploitation

Temp = Temp * Cooling_Factor;

END FOR

% 3. Output

Decode the global best position gbest;

Extract optimal feature subset F_best and optimal hyperparameters H_best;
