# Supplementary material for: Development of an automated machine learning-based risk prediction and decision support system for postoperative cubitus varus complicating pediatric lateral humeral condyle fracture
Source: Front Surg. 2026 Jul 30;13:1836372. doi: 10.3389/fsurg.2026.1836372 (PMC13469642; doi:10.3389/fsurg.2026.1836372)
Supplement: Supplementary file 2 [file Supplementaryfile2.docx]

**Supplementary materials 2**

**Statistical Tests of Swarm Intelligence Algorithms on CEC2022 Unimodal Benchmark Functions**

**1. Overall Validation: Friedman and Iman–Davenport Tests**

With the test functions serving as blocks and the eight algorithms as treatments, a non-parametric Friedman test was performed on the minimization objective function values (error; smaller values indicate superior performance). The distribution characteristics (median, interquartile range, and outliers) of the 30 independent-run errors for each algorithm on each test function are comprehensively presented as box plots in Figure 2A of the main manuscript; to avoid redundancy, these descriptive statistics are not duplicated here in tabular form. The Friedman test yielded a statistic of χ² = 51.11 (number of algorithms k = 8, number of blocks N = 12, degrees of freedom = 7) with p = 8.74e-09, indicating that the performance differences among the eight algorithms jointly considering all test functions are statistically significant. Application of the Iman–Davenport correction to the Friedman statistic gave F = 17.09 (df₁ = 7, df₂ = 77), with p = 1.95e-13, confirming the significant conclusion.

**2. Post-Hoc Paired Comparisons: ILEE versus the Remaining Seven Algorithms**

Building upon the overall validation that demonstrated significant differences among the eight algorithms, the ILEE algorithm was designated as the control. For each test function, the mean error of the 30 runs was calculated for every algorithm, and then the paired differences dⱼ = (mean error of ILEE)ⱼ − (mean error of the comparison algorithm)ⱼ were computed, yielding 12 paired differences across the test functions. A two-sided Wilcoxon signed-rank test was conducted on the d₁–d₁₂ values, and Holm’s sequential correction was applied to all seven pairwise comparisons (ILEE vs. LEE, WOA, GWO, PSO, GA, GA-PSO, and GA-ACO, respectively). After correction, all seven comparisons were significant at α = 0.05 (all pHolm < 0.05); that is, none of the seven comparison algorithms were superior to ILEE in terms of the comprehensive paired differences over the 12 functions. Detailed results are presented in Table S1.

**Table S1. Post-hoc paired comparisons between ILEE and the other seven algorithms**

| **Comparison** | **Number of blocks N** | **pHolm** | **Reject null at α = 0.05?** |
| --- | --- | --- | --- |
| ILEE vs LEE | 12 | 0.004 | Yes |
| ILEE vs WOA | 12 | 0.003 | Yes |
| ILEE vs GWO | 12 | 0.005 | Yes |
| ILEE vs PSO | 12 | 0.003 | Yes |
| ILEE vs GA | 12 | 0.004 | Yes |
| ILEE vs GA-PSO | 12 | 0.004 | Yes |
| ILEE vs GA-ACO | 12 | 0.021 | Yes |

Note: pHolm denotes the adjusted p-value after Holm’s sequential correction. All comparisons rejected the null hypothesis, indicating that the overall optimization error of ILEE is significantly lower than that of the comparison algorithms.

**3. Win/Tie/Loss Results of Two-Sample Tests per Test Function**

For each individual test function, the errors from the 30 independent runs of each pair of algorithms were compared using a two-independent-sample Mann–Whitney U test (two-sided, nominal α = 0.05, without pairing over run indices). This covered all seven comparison algorithms other than ILEE. When p < 0.05, the algorithm with the superior median error was deemed better; when p ≥ 0.05, the result was recorded as a tie. A “win” indicates that the difference was significant at the nominal α = 0.05 level and the median error of ILEE was superior; a “loss” indicates that the comparison algorithm was significantly superior. The results are summarized in Table S2.

**Table S2. Per-function two-sample test results**

| **Comparison** | **Wins (ILEE)** | **Ties** | **Losses** |
| --- | --- | --- | --- |
| ILEE vs LEE | 11 | 1 | 0 |
| ILEE vs WOA | 12 | 0 | 0 |
| ILEE vs GWO | 8 | 3 | 1 |
| ILEE vs PSO | 12 | 0 | 0 |
| ILEE vs GA | 10 | 2 | 0 |
| ILEE vs GA-PSO | 9 | 2 | 1 |
| ILEE vs GA-ACO | 9 | 2 | 1 |
